# Clinical features and risk factors for severe and critical pregnant women with 2009 pandemic H1N1 influenza infection in China

**DOI:** 10.1186/1471-2334-12-29

**Published:** 2012-02-01

**Authors:** Peng-jun Zhang, Xiao-li Li, Bin Cao, Shi-gui Yang, Li-rong Liang, Li Gu, Zhen Xu, Ke Hu, Hong-yuan Zhang, Xi-xin Yan, Wen-bao Huang, Wei Chen, Jing-xiao Zhang, Lan-juan Li, Chen Wang

**Affiliations:** 1 Beijing Chao-Yang Hospital, Beijing Institute of Respiratory Medicine, Beijing Key Laboratory of Respiratory and Pulmonary Circulation Disorders, Department of Respiratory Medicine, Capital Medical University, Beijing, China; 2 State Key Laboratory for Diagnosis and Treatment of Infectious Diseases, The First Affiliated Hospital, School of Medicine, Zhejiang University, Key Laboratory of Infectious Diseases Key Laboratory of Infectious Diseases, Zhejiang University, Hangzhou, China; 3 Disease Control and Emergency Response Office, Chinese Center for Disease Control and Prevention, Beijing, China; 4 Renmin Hospital of Wuhan University, Wuhan, China; 5 The First Affiliated Hospital of Anhui medical university, Hefei, China; 6 Department of Respiratory, Second Hospital of Hebei Medical University, Shi Jiazhuang, China; 7 Hang zhou No.1 People's hospital, Hangzhou, China; 8 Shengjing Hospital of China Medical University, Shenyang, China; 9 the Second Affiliated Hospital, Nanchang University, Changchun, China; 10 Department of Respiratory Medicine, Capital Medical University, Beijing Institute of Respiratory Medicine, Beijing Key Laboratory of Respiratory and Pulmonary Circulation Disorders, Beijing Hospital, Ministry of Heath, Beijing, China

**Keywords:** Pregnant women, Neonate, Pandemic H1N1 influenza, Mortality, Non-invasive ventilation

## Abstract

**Background:**

2009 pandemic H1N1 (pH1N1) influenza posed an increased risk of severe illness among pregnant women. Data on risk factors associated with death of pregnant women and neonates with pH1N1 infections are limited outside of developed countries.

**Methods:**

Retrospective observational study in 394 severe or critical pregnant women admitted to a hospital with pH1N1 influenza from Sep. 1, 2009 to Dec. 31, 2009. rRT-PCR testing was used to confirm infection. In-hospital mortality was the primary endpoint of this study. Univariable logistic analysis and multivariate logistic regression analysis were used to investigate the potential factors on admission that might be associated with the maternal and neonatal mortality.

**Results:**

394 pregnant women were included, 286 were infected with pH1N1 in the third trimester. 351 had pneumonia, and 77 died. A PaO_2_/FiO_2 _≤ 200 (odds ratio (OR), 27.16; 95% confidence interval (CI), 2.64-279.70) and higher BMI (i.e. ≥ 30) on admission (OR, 1.26; 95% CI, 1.09 to 1.47) were independent risk factors for maternal death. Of 211 deliveries, 146 neonates survived. Premature delivery (OR, 4.17; 95% CI, 1.19-14.56) was associated neonatal mortality. Among 186 patients who received mechanical ventilation, 83 patients were treated with non-invasive ventilation (NIV) and 38 were successful with NIV. The death rate was lower among patients who initially received NIV than those who were initially intubated (24/83, 28.9% vs 43/87, 49.4%; *p *= 0.006). Septic shock was an independent risk factor for failure of NIV.

**Conclusions:**

Severe hypoxemia and higher BMI on admission were associated with adverse outcomes for pregnant women. Preterm delivery was a risk factor for neonatal death among pregnant women with pH1N1 influenza infection. NIV may be useful in selected pregnant women without septic shock.

## Background

Pregnant women are at an increased risk for contracting influenza and its complications associated with influenza [1]. Like previous epidemic and pandemic diseases, 2009 pandemic H1N1 (pH1N1) influenza posed an increased risk of severe illness among pregnant women [2-9]. A report from the first month of the pH1N1 outbreak noted that the rate of hospitalization among pregnant women was approximately four times the rate in the general population in the USA [3]. As reported by the California Department of Public Health (CDPH), a total of 10% of the 1088 patients who were hospitalized or died from the 2009 pH1N1 influenza were pregnant [10]. According to the Ministry of Health (MOH) of the People's Republic of China, pregnant women accounted for 13.7% of deaths associated with 2009 pH1N1 influenza [11]. Pregnant women with influenza appear to have an increased risk of miscarriage, premature birth and stillbirth [2,12,13]. Reports from Victoria in Australia [14,15], New York [16], and California [17], demonstrate that 2009 pH1N1 infection was associated with substantial maternal and fetal morbidity and mortality. However, information is limited concerning the risk factors for maternal and neonatal death when pregnancy is complicated by severe or critical illness related to 2009 pH1N1 influenza.

In this report, we described the characteristics of pH1N1 influenza in pregnant women and the risk factors for maternal and neonatal death.

## Methods

### Study patients

All patients who were admitted to hospitals with confirmed 2009 pH1N1 influenza from Sep. 1 to Dec. 31, 2009 from 27 Chinese provinces were screened if they fulfilled the diagnostic criteria for severe or critical cases. A confirmed case was a person whose pH1N1 virus infection was verified by real-time reverse-transcriptase polymerase chain reaction (rRT-PCR) with or without the presentation of other clinical symptoms. Patients were excluded if they had been treated as outpatients or in emergency rooms or duration of hospitalization < 24 h, or if they had incomplete records of clinical outcomes. Severe and critical cases were defined according to the H1N1 2009 Clinical guidelines (Third Edition, 2009) released by the MOH (Additional file 1: Table S1). Our research retrospectively collected the patient's clinical information and did not involve the patient's personal information and samples, so there was no informed consent.

### Study design and data collection

The case report form included demographic information, underlying conditions, gestational age, vaccination status, treatment, intensive care unit (ICU) admission, complications, and maternal and neonatal outcomes. Body mass index (BMI) was calculated using height and weight recorded in the case report form, patients with BMI ≥ 30 were categorized as obesity. Indications for applying non-invasive ventilation (NIV): pregnant women who complained shortness of breath or blood gas analysis confirmed hypoxemia PaO_2 _to FiO_2 _< 300. One non-pulmonary major organ dysfunction or unconsciousness was contraindications for NIV. Indications to change from NIV to invasive ventilation: A cautious trial of NIV was attempted and response to NIV was monitored after the first hour or two. If there was a deterioration of oxygenation, invasive ventilation was considered. Definition of successful NIV: PaO_2 _to FiO_2 _improved and respiratory rate decreased during one or two hour NIV therapy. The patients successfully weaned off NIV and survived. Definition of failed NIV: During the one or two NIV trial, a deterioration of oxygenation was observed and invasive ventilation was needed. Data collection and analysis were coordinated by the MOH. A standard data collection form was used for each study site. Site investigators were primarily infectious disease physicians closely involved in taking care of such patients at their centers. The data were entered in duplicate into a computerized database. Patient confidentiality was maintained by recording only patient date of birth and gender on the data collection form. The research ethics board at Beijing Chao-Yang Hospital and The First Affiliated Hospital, School of Medicine, Zhejiang University approved the study.

### Analysis

We analyzed the reported demographic characteristics, underlying conditions, symptoms, treatments, complications, clinical course and maternal and neonatal outcomes. Means (standard deviations, SD) or medians (interquartiles, IQR) were calculated as summaries of continuous variables. For categorical variables, percentages of patients in each category were calculated. We compared clinical characteristics and clinical outcomes by using an ANOVA test, chi-square test, or Fisher's exact test or Wilcoxon rank-sum test as necessary. The primary outcome was in-hospital mortality. We performed univariable logistic analysis to investigate the potential factors on admission that might be associated with the maternal mortality. Factors with statistical significance (*p *< 0.05) in the univariate analyses were included in the multivariate logistic regression analysis. A *p *value of less than 0.05 was considered to indicate statistical significance. All analysis was carried out using SPSS for Windows (release 13.0).

## Results

### Clinical description of cohort

**Figure 1:**
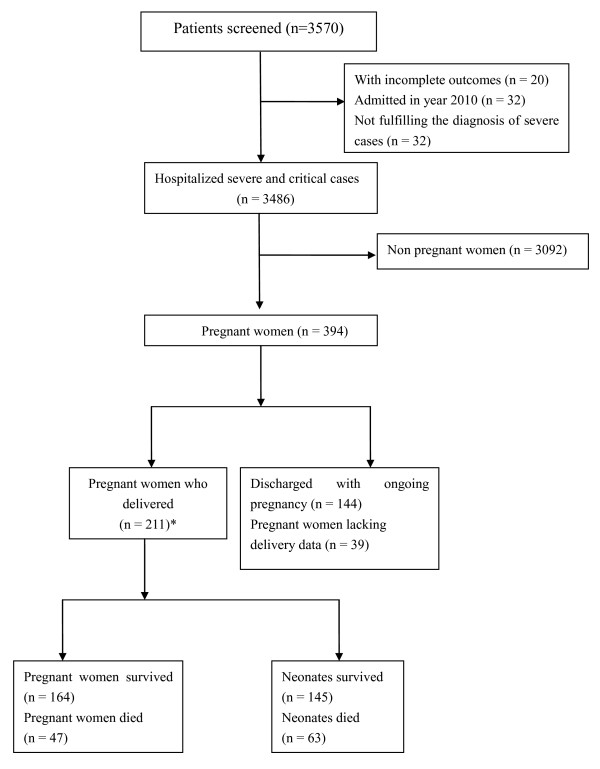
Flow chart of patients enrolled and included in the analysis * Missing data for neonatal outcomes (n = 3).

3570 severe or critical cases were screened and 394 cases involved pregnant women (Figure 1). Demographic characteristics, underlying conditions, symptoms, and lab findings of the 394 pregnant women are illustrated in Table 1. The median age was 25.0 years old (IQR 23.0 to 28.0) and 21 patients (5.6%) were more than 35 years old. Of all available data, 35 patients (14.4%) had a BMI of more than 30 and 10 patients (4.1%) had a BMI of more than 35. The median gestational age was 32 weeks (IQR, 26 to 37), with 72.6% of patients in the third trimester. Eleven patients (2.8%) had respiratory diseases and thirteen (3.3%) had cardiovascular diseases. Other coexisting diseases were rare in this analysis. None of the patients had been immunized against seasonal influenza or 2009 pH1N1. The median APACHE II score was 7.0 (IQR, 4-11). At the time of admission, 351 patients (90.0%) had pneumonia with an abnormal chest radiography or chest computed tomography. The most common symptoms were cough (372; 94.7%) and dyspnoea (199; 50.6%). The median PaO_2_/FiO_2 _on admission was 154.7 (IQR, 89.5-320.5) (Table 1). Of the 394 hospitalized patients, 246 (63.7%) were admitted to an ICU at a median of 8 days from onset of illness (IQR 5 to 14; Table 2).

**Table 1:** Demographic characteristics, underlying conditions, symptoms and lab findings of 394 pregnant patients*

Variables	Value
Age, years	

median years (IQR)	25 (23-28)

≤ 20 y (%)	23/375 (6.1)

20-35 y (%)	331/375 (88.3)

> 35 y (%)	21/375 (5.6)

Han Chinese (%)	371/393 (94.4)

Job	

Farmer (%)	200/389 (51.4)

Unemployed (%)	84/389 (21.6)

Others (%)†	105/389 (27.0)

BMI, median (IQR)	26.0 (23.4-29.3)

≤ 25.0 (%)	99/243 (40.7)

25-30 (%)	99/243 (40.7)

30-35 (%)	35/243 (14.4)

> 35 (%)	10/243 (4.1)

Gestational age§	

1^st ^trimester (0-14 week) (%)	20/394 (5.1)

2^nd ^trimester (15-27 week) (%)	88/394 (22.3)

3^rd ^trimester (≥ 28 week) (%)	286/394 (72.6)

**Chronic pre-existing disease**	

Respiratory diseases** (%)	11/388 (2.8)

Cardiovascular diseases*** (%)	13/393 (3.3)

Diabetes mellitus (%)	4/394 (1.0)

Cancer or hematological diseases (%)	3/394 (0.8)

Immune suppressed (%)	1/390 (0.3)

Pneumonia (%)	351/390 (90.0)

**Symptoms and Lab findings**	

T ≥ 38°C (%)	340/374 (90.9)

Cough (%)	372/393 (94.7)

Dyspnoea (%)	199/393 (50.6)

Hemoptysis (%)	55/392 (14.0)

Pleural effusion (%)	44/226 (19.5)

CNS symptom ※ (%)	48/392 (12.2)

WBC (×10^9^/L)	7.4 ± 3.7

Platelet (×10^9^/L)	169.0 ± 65.7

CK > 200 u/L (%)	77/306 (19.5)

CRP (mg/L)	42 (16-101)

ESR (mm/h)	35 (23-50)

PaO_2_/FiO_2_, median (IQR)	154.7 (89.5-320.5)

APACHE II scores, median (IQR)	7 (4-11)

IQR, interquartile range; BMI, body mass index; APACHE II, acute physiology and chronic health evaluation II

* Data are presented as no./total no., if otherwise stated. Percentages are based on women with complete information in the respective categories

Nineteen patients missed the detailed information. Percentages may not total 100 because of rounding

† Other job included student (in two patients), teacher (in twelve), babysitter (in one), catering (in one), business service (in eleven), worker (in nine), migrant laborer (in ten), herdsman (in one), professional staff member (in seventeen), retired (in one), health care worker (in three), other detailed (in thirty-seven)

§ The median gestational age was 32 weeks (IQR, 26-37 weeks)

** included asthma, COPD, active tuberculosis and other bronchial disease

***included coronary heart disease, chronic congestive heart failure, valvular disease

※ CNS system symptoms: refers to one or more of the following symptoms: insomnia, restlessness, hallucination, headache, dizziness and abnormal behaviour

**Table 2:** Treatment, complications, outcomes among pregnant women with confirmed 2009 pH1N1 influenza and neonatal outcomes*

Variables	Value
**Treatment**	

Antibiotics (%)	387/394 (98.2)

Corticosteroids (%)	242/394 (61.4)

Mechanical ventilation** (%)	186/394 (47.2)

Non-invasive ventilation, successful(%)	38/170 (22.4)

Non-invasive ventilation, failed (%)	45/170 (26.5)

Initially intubated (%)	87/170 (51.1)

Oseltamivir (%)	378/394 (95.9)

Interval from onset to oseltamivir ≤ 48 h (%)	52/371 (14.0)

Interval from onset to oseltamivir > 48 h (%)	319/371 (86.0)

Traditional Chinese Medicine (%)	244/392 (62.2)

**Complications**	

ARDS (%)	151/283 (53.4)

Septic shock (%)	51/254 (20.1)

Acute renal failure (%)	11/237 (4.6)

Acute liver damage‡ (%)	73/260 (28.1)

DIC (%)	8/234 (3.4)

**Clinical course and Maternal Outcomes**	

ICU admission (%)	246/386 (63.7)

Length of ICU stay, median (IQR)	8 (5-14)

Time from onset to fever clearance, median (IQR)	7 (5-10)

Hospital stay for survivors, median (IQR)	11 (7-17)

In-hospital mortality (%)	77/394 (19.5)

Time from onset to death, median (IQR)	11 (7-17)

**Delivery methods**	

Vaginal delivery (%)	31/208 (14.9)

Vacuum-assisted vaginal delivery (%)	2/208 (1.0)

Forceps-assisted vaginal delivery (%)	3/208 (1.4)

Cesarean delivery (%)	172/208 (82.7)

**Neonatal outcomes**	

Survival	146/208 (70.2)

Stillbirth	36/208 (17.3)

Neonatal death	12/208 (4.8)

Spontaneous abortion	1/208 (0.5)

Therapeutic abortion	13/208 (6.3)

ARDS, acute respiratory distress syndrome; DIC, disseminated intravascular coagulation; ICU, intensive care unit; IQR, interquartile range.

* Date are presented as no./total no. if otherwise stated. Percentages are based on women with complete information in the respective categories.

** Sixteen patients missed the strategies of mechanical ventilation.

¶¶Acute renal failure: Serum Creatinine increased by 2-fold or GFR decreased > 50%, or urine   0.5 ml/kg/h for at least 12 h.

‡ Acute liver damage: AST or ALT > 70 U/L, or Tbil > 2 mg/dL

### Medication

378 (95.9%) patients received oseltamivir. The median time from onset of illness to oseltamivir therapy was 5 days (IQR 3 to 7), among them only 52 patients (14.0%) received oseltamivir within 48 h of onset of illness. 387 out of 394 patients received antibiotics. 244 received traditional Chinese medicine. Corticosteroid therapy was administered to 242 patients (Table 2).

### Maternal and neonatal outcomes

The most commonly reported complication in this study was acute respiratory disease syndrome (ARDS) (151; 53.4%) (Table 2). 211 (59.4%) women delivered at a median of 6 days (IQR 3 to 12) after pH1N1 symptom onset. 122 out of 211 women delivered prematurely (Additional file 2: Table S2). The most common delivery method was cesarean delivery (172 patients, 82.7%) (Table 2). Among 143 live-birth deliveries for which the gestational age was known, 68 were premature (Additional file 2: Table S2). Among the 394 pregnant women in the study, 77 died (Table 2), 56 out of the 77 patients who died were in their third trimester. The main cause of death was refractory hypoxemia (66 patients, 85.7%). Of 5 patients with secondary infection, three patients had *Acinetobacter baumannii*, one patient had *Aspergillus spp*, and one patient had both *Acinetobacter baumannii *and *Aspergillus spp*.

### Mechanical ventilation

62.4% of women included in the study required intensive care and 47.2% required mechanical ventilation. 83 patients received NIV and 38 patients succeeded with NIV. Among 45 patients who failed with initial NIV, 38 of them were then administered invasive ventilation, and 24 of 38 these patients died. The death rate was lower among patients who initially received NIV than those were initially intubated (24/83, 28.9% vs 43/87, 49.4%; *p *= 0.006).

Univariate analyses showed that patients who failed NIV treatment had higher APACHE II scores (OR, 1.14; 95% CI, 1.02 to 1.27; *p *= 0.01), more CNS symptoms (OR, 9.51; 95% CI, 1.15 to 79.03; *p *= 0.04), septic shock (OR, 27.93; 95% CI, 3.34 to 33.47; *p *= 0.002), and a higher incidence of acute liver damage (OR, 3.93; 95% CI, 1.07 to 14.52; *p *= 0.04) compared with those who succeeded with NIV therapy. Multivariable analyses suggested that pregnant women with pH1N1 virus complicated by septic shock (OR, 19.23; 95% CI, 1.97 to 187.13; *p *= 0.011) were less likely to be successfully treated by NIV (Table 3).

**Table 3:** Comparison between patients who succeeded with non-invasive ventilation and those who did not*

Characteristic	Non-invasive ventilation				
	
	With successful outcomes‡ (n = 38)	With failure outcomes (n = 45)	*P *value	OR_uadj _(95% CI)	OR_adj _(95% CI)
Age (Mean ± SD, years)	26.1 ± 4.3	25.5 ± 4.3	0.61	0.97 (0.88-1.08)	

BMI, median (IQR)	25.2 (24.4-28.8)	27.0 (24.5-30.4)	0.31	1.08 (0.93-1.25)	1.06 (0.72-1.56)

Gestational age, median (IQR)	32 (26-35)	32 (28-36)	0.96	0.99 (0.93-1.07)	0.97 (0.82-1.15)

Respiratory diseases (%)	2/37 (5.4)	1/44 (2.3)	0.48	0.42 (0.04-4.79)	

Pneumonia (%)	37/38 (97.4)	43/45 (95.6)	0.65	1.76 (0.15-20.23)	

**Symptoms and Lab findings**					

T ≥ 38°C(%)	33/37 (89.2)	39/42 (92.9)	0.86	1.57 (0.33-7.56)	

Dyspnoea (%)	24/38 (63.2)	31/44 (70.5)	0.43	0.68 (0.26-1.76)	

Hemoptysis (%)	6/38 (15.8)	9/43 (20.9)	0.62	1.34 (0.42-4.23)	

CNS symptom ※ (%)	1/38 (2.6)	9/44 (20.5)	0.04	9.51 (1.15-79.03)	1.48 (0.96-13.45)

WBC (×10^9^/L), (Mean ± SD)	7.2 ± 3.6	7.6 ± 3.9	0.63	1.03 (0.91-1.16)	

Hb (g/L) (Mean ± SD)	105.9 ± 15.6	99.0 ± 15.7	0.06	0.97 (0.94-1.02)	

PLT (×10^9^/L) (Mean ± SD)	155.5 ± 44.1	151.7 ± 56.7	0.75	1.00 (0.99-1.01)	

ALT (U/L) (Mean ± SD)	43.3 ± 60.9	48.9 ± 61.2	0.69	1.002 (0.99-1.01)	

CRP (mg/L) (Mean ± SD)	71.2 ± 52.9	90.8 ± 56.6	0.36	1.01 (0.99-1.02)	

PaO_2_/FiO_2_					

PaO_2_/FiO_2 _≥ 300 (%)	7/24 (29.2)	3/33 (9.1)		Referent	

PaO_2_/FiO_2 _201 to 300 (%)	3/24 (12.5)	4/33 (12.1)	0.27	3.11 (0.41-23.93)	

PaO_2_/FiO_2 _≤ 200 (%)	14/24 (58.3)	26/33 (78.8)	0.06	4.33 (0.97-23.83)	

APACHE II score, median (IQR)	7 (7-11)	10 (6-14)	0.01	1.14 (1.02-1.27)	1.20 (0.97-1.49)

Septic shock (%)	1/24 (4.2)	17/32 (53.1)	0.002	27.93 (3.34-33.47)	32.87 (1.91-566.52)

Acute liver damage **(%)	4/25 (16.0)	12/28 (42.9)	0.04	3.93 (1.07-14.52)	5.17 (0.47-56.37)

Pleural effusion (%)	11/35 (31.4)	12/40 (30.0)	0.89	1.07 (0.40-2.86)	

OR_uadj_, unadjusted odds ratio; OR_adj_, Adjusted odds ratio; CI, confidence Interval; BMI, body mass index; IQR, interquartile range; APACHE II, acute physiology and chronic health evaluation II; ICU, intensive care unit

* Date are presented as no./total no., if otherwise stated. Percentages are based on women with complete information in the respective categories

‡ included 38 patients survived after receiving NIV

¶ included 7 patients died of respiratory failure who received NIV, 38 patients who received NIV firstly, then changed to invasive ventilation

※ CNS system symptoms: refers to one or more of the following symptoms: insomnia, restlessness, hallucination, headache, dizziness and abnormal behaviour.

** Acute liver damage: AST or ALT > 70 U/L, or Tbil > 2 mg/dL.

Variables included in multivariate model: BMI, Gestational age, CNS symptom, APACHE II score, septic shock, acute liver damage

### Risk factor for maternal and neonatal death

62 out of 208 births resulted in neonatal death. 118 out of 208 births were premature. A multivariate analysis was applied to investigate the factors associated with pregnant outcomes. A PaO_2_/FiO_2 _≤ 200 on admission (OR, 27.16; 95% CI 2.64 to 279.70, *p *< 0.001) and higher BMI (i.e. ≥ 30) on admission (OR, 1.26; 95% CI 1.09 to 1.47, *p *= 0.86) were independent risk factors for maternal death (Table 4). Premature delivery (OR, 4.17; 95% CI 1.19 to 14.56, *p *< 0.001) was associated with neonatal death (Table 5).

**Table 4:** Univariate and multivariate regression analysis of the association of factors with death among pregnant women with confirmed pH1N1 influenza*

Variables	Patients who survived (n = 317)	Patients who died (n = 77)	*P *value	OR_uadj _(95% CI)	OR_adj _(95% CI)
Age, median years (IQR)	24.8 (22.9-28.3)	23.9 (21.7-28.3)	0.10	0.95 (0.90-1.01)	

BMI, median (IQR)	25.99 (23.20-29.26)	27.37 (24.87-30.33)	0.02	1.10 (1.01-1.17)	1.26 (1.09-1.47)

Gestational age, median (IQR)	32 (26-37)	32 (26-38)	0.86	0.97 (0.97-1.03)	0.94 (0.86-1.03)

Comorbidity **(%)	25/317 (7.9)	6/77 (7.8)	0.97	0.98 (0.39-2.50)	

Pneumonia (%)	275/311 (88.4)	75/77 (97.4)	0.03	4.91 (1.16-20.86)	0.68(0.02-2.39)

**Symptoms and Lab findings**					

PaO_2_/FiO_2_, median (IQR)					

PaO_2_/FiO_2 _≥ 300 (%)	62/164 (37.8)	1/55 (1.8)		Referent	Referent

PaO_2_/FiO_2 _201 to 300 (%)	23/164 (14.0)	2/55 (3.6)	0.18	5.39 (0.47-62.33)	2.05 (0.09-46.80)

PaO_2_/FiO_2 _≤ 200 (%)	79/164 (48.2)	52/55 (94.5)	< 0.001	40.81 (5.49-303.51)	27.16 (2.64-279.70)

APACHE II score, median (IQR)	6 (3-10)	12 (8-16)	< 0.001	1.13 (1.09-1.18)	1.07 (0.98-1.18)

Dyspnoea (%)	139/315 (44.1)	58/76 (76.3)	< 0.001	4.08 (2.30-7.24)	1.81 (0.47-7.05)

Hemoptysis (%)	33/315 (10.5)	21/75 (28.0)	< 0.001	3.32 (1.79-6.18)	1.64 (0.37-7.27)

CNS symptom (%)	29/314 (9.2)	19/76 (25.0)	< 0.001	3.27 (1.71-6.24)	2.99 (0.63-14.14)

AST (u/L), median (IQR)	35 (23-65)	96.10 (44.50-159.5)	< 0.001	1.007 (1.004-1.011)	1.00 (0.99-1.00)

OR, Odds Ratio; CI, confidence Interval; OR_uadj_, unadjusted odds ratio; OR_adj_, Adjusted odds ratio; BMI, body mass index; IQR, interquartile range; APACHE II, acute physiology and chronic health evaluation II

* Data are presented as no./total no., if otherwise stated. Percentages are based on women with complete information in the respective categories

Variables included in univariate model: Age, BMI, Gestational age, comorbidity, pneumonia and symptoms and lab findings on admission associated with mortality

Variables included in multivariate model: BMI, Gestational age, PaO_2_/FiO_2 _on admission, APACHE II score, dyspnoea, hemoptysis, CNS symptom, AST

** Comorbidity included Respiratory diseases (asthma, COPD, active tuberculosis and other bronchial disease), cardiovascular diseases (heart disease, chronic congestive heart failure, valvular disease), diabetes mellitus, cancer or hematological diseases and Immune suppressed

**Table 5:** Univariate and multivariate analysis of the association of maternal characteristics with death among neonates*

Variables	Neonates who survived (n = 145)	Neonates who died (n = 63)	*P *value	OR_uadj _(95% CI)	OR_adj _(95% CI)
Maternal BMI, median (IQR)	27.5 (25.0-30.1)	25.6 (23.0-29.4)	0.06	0.91 (0.83-1.00)	1.05 (0.91-1.23)

Maternal Age, median years (IQR)	24.4 (20.9-27.9)	25.3 (22.5-28.6)	0.57	1.02 (0.96-1.08)	

Gestational age					

Non-premature delivery (≥ 37 weeks)	76/145 (52.4)	12/63 (19.0)		Referent	Referent

Preterm delivery (< 37 weeks)	69/145 (47.6)	51/63 (81.0)	< 0.001	4.68 (2.31-9.51)	4.17 (1.19-14.56)

Comorbidities ** (%)	16/145 (11.0)	3 (4.8)	0.15	0.40 (0.11-1.44)	

**Symptoms and Lab findings**					

CNS symptom (%)	10/145 (6.9)	13/63 (20.6)	0.005	3.51 (1.45-8.51)	0.85 (0.08-9.47)

AST (u/L), median (IQR)	40 (26-76)	73 (36-139)	0.01	1.00 (1.00-1.01)	1.00 (0.99-1.01)

PaO_2_/FiO_2 _on admission					

PaO_2_/FiO_2 _≥ 300 (%)	61/161 (37.9)	2/58 (3.4)		Referent	Referent

PaO_2_/FiO_2 _201 to 300 (%)	22/161 (13.7)	3/58 (5.2)	0.13	4.16 (0.65-26.57)	3.59 (0.11-121.48)

PaO_2_/FiO_2 _≤ 200 (%)	78/161 (48.4)	53/58 (91.4)	< 0.001	20.72 (4.86-88.44)	12.33 (0.86-177.49)

APACHE II score, median (IQR)	8 (5-12)	10 (5-14)	0.17	1.04 (0.98-1.09)	0.99 (0.87-1.14)

OR, Odds Ratio; CI, confidence Interval; OR_uadj_, unadjusted odds ratio; OR_adj_, Adjusted odds ratio; BMI, body mass index; IQR, interquartile range; APACHE II, acute physiology and chronic health evaluation II; ARDS, acute respiratory distress syndrome

* Date are presented as no./total no., if otherwise stated. Percentages are based on women with complete information in the respective categories

** Comorbidities included respiratory diseases (asthma, COPD, active tuberculosis and other bronchial disease), cardiovascular diseases (heart disease, chronic congestive heart failure, valvular disease), diabetes mellitus, cancer or hematological diseases and Immune suppressed

Values in the multivariate analysis included maternal BMI, gestational age, CNS symptom, AST, APACHE II score and PaO_2_/FiO_2 _on admission

## Discussion

The first case of 2009 pH1N1 virus infection in China was documented on May 10, the virus has rapidly spread throughout the mainland. A total of 126,000 confirmed cases were reported by Mar 31, 2010, including 7414 patients severe and 800 patients died. Among all these severe cases, about 13.7% of patients were pregnant women [18].

In this large study of pregnant women who were hospitalized with severe 2009 pH1N1 influenza, the clinical characteristics were similar to those reported by others [3,4,17,19]. 95.6% of patients were infected in the second or third trimester. In our study, the most common comorbidities were cardiovascular diseases (3.3%), diabetes mellitus (1.0%), respiratory diseases (2.8%), and obesity (18.5%). In our study, the prevalence of underlying diseases was much lower than reports from the United States (49.3%) [19], 56% in Australia [14], 34% in California [17], 22.8% in Brazil [20], and 62% in France [4]. In those studies, the main cause of underlying disease was asthma. A study compared asthma prevalence of Chinese adolescents living in Canada and in China. The authors found that for girls, the range of asthma was 4.3% in Guangzhou to 9.8% in Canadian-born Chinese adolescents. These results suggest that the lower prevalence of pre-existing asthma in our samples reflects prevalence of the disease in the Chinese population [21].

The mortality rate for severe or critically infected pregnant women in our study was 20%, similar to what was reported in Canada, Mexico, and New Zealand [22-25], but higher than in France (8% death in ICU-hospitalized pregnancy women) [4]. Risk analysis showed that a PaO_2_/FiO_2 _≤ 200 and higher BMI (i.e. ≥ 30) on admission were risk factors for maternal death. Pregnancy and ARDS are associated with increased oxygen consumption, which can result in hypoxemia in the mothers and the neonates. We reported that a higher BMI was associated with maternal mortality after adjusting for baseline clinical factors. Observations of a high prevalence of obesity in severe and fatal cases of 2009 pH1N1 infection have been reported in Chile, Canada, the United Kingdom and Mexico [10,26,27]. As observed in Australia, 42% of patients had a BMI of more than 30 and 22% of patients more than 35, while the corresponding proportions in the general Australian pregnant population was 24% and 10% respectively [28]. However, our research retrospectively collected the patient's clinical information recorded in CRFs. Proportion of obesity has been overestimated based on BMI in the 3^rd ^trimester of pregnancy.

Data from previous pandemics and seasonal influenza epidemics suggested that the risk of complications associated with influenza might be higher in the second and third trimester of pregnancy than in the first trimester [2,3,17]. We also observed a higher proportion of maternal death occurring in the second and third trimester. During the 2009 H1N1 influenza pandemic, in the United States, the rate of premature birth (30.2%) was higher than the rate of premature births (13%) reported in 2007 [29], consistent with data demonstrating a higher rate of premature delivery during previous pandemics [2]. Among women in our study for whom data on pregnancy outcomes was available, the rate of premature birth was 57.8%. In a multivariable analysis, preterm delivery contributed to fetal mortality. Delivery in severe and critically infected women after 37 weeks' of gestation had improved neonatal outcomes compared to similar patients who delivered before 37 weeks of gestation.

Evidence on the useful role of NIV in pregnant patients with ARDS secondary H1N1 viral infection was lacking. Dr. Amit Banga [30] reported a 28-year-old pregnant female with ARDS (PaO_2_/FiO_2 _155) due to community-acquired severe pneumonia who successfully treated with NIV. In 2009, Dr. Michel Djibre and collegues [31] reported a 38-year-old pregnant woman at 31 weeks' gestation with PaO_2_/FiO_2 _98 who was successfully treated with NIV. In our study, the success rate among pregnant women with H1N1 infection for NIV was 45.8%. A recent prospective multicenter survey also found that when NIV was used as first-line therapy for selected ALI/ARDS patients (those with 2 organ failures, hemodynamic instability, or encephalopathy were excluded), 54% avoided intubation and had excellent outcomes [32].

Apart from previous findings that major organ dysfunction and obtunded sensorium would obviously be unsuitable candidates for NIV, we found that pregnant women complicated by septic shock were less likely to be successfully treated by NIV. Our data also support that cautious selection of appropriate patients is important for successful application of NIV. Patients should be monitored closely for signs of NIV failure until stabilized. If there are signs of NIV failure, patients should be intubated promptly before a crisis develops.

Our investigation has several limitations. Firstly, we only evaluated pregnant women admitted to a hospital who fulfilled the diagnostic criteria of severe or critical cases. Secondly, it was an observational study, and could therefore only demonstrate associations and could not infer cause. Thirdly, we lacked follow up visits for maternal and neonatal outcomes. Lastly, despite the use of a standardized data-collection form, not all information was collected for all patients.

## Conclusions

The clinical data reported herein is consistent with previous studies that demonstrate that pregnant women with influenza are at an increased risk of serious illness and death. Our novel findings included: 1) NIV was useful for some selected pregnant women with pH1N1 virus infection complicated by respiratory failure, but septic shock should be considered a contraindication; 2) a PaO_2_/FiO_2 _≤ 200 and higher BMI (i.e. ≥ 30) on admission were independent risk factors for maternal death; 3) Premature delivery was an independent risk factor for neonatal death.

## Abbreviations

NIV: Non-invasive ventilation; pH1N1: Pandemic H1N1; APACHE II: Acute Physiology and Chronic Health Evaluation II; BMI: Body mass index; CDPH: California Department of Public Health; MOH: Ministry of Health; ARDS: Acute respiratory distress syndrome; rRT-PCR: Real-time reverse-transcriptase polymerase chain reaction; ICU: Intensive care unit; OR: Odds Ratio; SD: Standard deviations; IQR: Interquartile range; CI: Confidence Interval; ALI: Acute lung injury; ARF: Acute Renal Failure; DIC: Disseminated intravascular coagulation; OR_uadj_: Unadjusted odds ratio; OR_adj_: Adjusted odds ratio.

## Competing interests

The authors declare that they have no competing interests.

## Authors' contributions

All authors made substantial contributions to conception and design, acquisition of data, or analysis and interpretation of data, reviewed and approval of the final manuscript. Drs. PJZ, XLL, BC and SGY contributed equally to this article. CW and LJ L, the principal investigator, takes full responsibility for the integrity of the submission and publication, and was involved in the study design as part of the steering committee, had full access to all the data in the study and takes responsibility for the integrity of the data and the accuracy of the data analysis. Drs Z PJ, L XL, CB, Y SG had full access to all of the data in the study, and they take responsibility for the integrity of the data and the accuracy of the data analysis and draft of the manuscript. Drs L LR and GLwere involved in the study design as part of the Steering committee. Drs XZ, HK, Z HY, Y XX, H WB, CW, Z JX were responsible for the patient enrollment and the data collection.

## Pre-publication history

The pre-publication history for this paper can be accessed here:

http://www.biomedcentral.com/1471-2334/12/29/prepub

## Supplementary Material

Additional file 1 **The diagnosis criteria for severe and critical cases**. Click here for file

Additional file 2 **Maternal and neonatal outcomes by different delivery methods in different trimesters**. Data are presented as no. (%)/total no.(%), if otherwise stated. Percentages are based on patients with complete information in the respective categories. * Two patients missed the detailed information in maternal outcomes. Neonatal outcomes were unknown in four cases. ** One patient missed the detailed information in maternal outcomes. Neonatal outcomes were unknown in two cases. Click here for file
