# Supplementary material for: Clinical features and risk factors for severe and critical pregnant women with 2009 pandemic H1N1 influenza infection in China
Source: BMC Infect Dis. 2012 Feb 1;12:29. doi: 10.1186/1471-2334-12-29 (PMC3311613; doi:10.1186/1471-2334-12-29)
Supplement: Additional file 1 — The diagnosis criteria for severe and critical cases. [file 1471-2334-12-29-S1.DOC]

**Additional file 1 - The diagnosis criteria for severe and critical cases**

| Severe case  (met at least one of the following criteria on admission) | Critical case  (met at least one of the following criteria on admission) |
| --- | --- |
| 1. high fever (temperature ≥ 39℃) >3 days 2. serious coughing, purulent sputum, blood sputum or chest pain 3. respiratory rate ≥ 20 times/min with dyspnoea or cyanosis 4. mental disorder: slow reaction, drowsiness or convulsion 5. serious vomiting, diarrhoea with dehydration 6. new radiographic abnormality indicating pneumonia (on chest radiograph or CT scan) 7. deterioration of underlying diseases | 1. respiratory failure 2. septic shock 3. multiple organs insufficiency 4. other severe clinical conditions requires intensive care |
