# Supplementary material for: Clinical features and risk factors for severe and critical pregnant women with 2009 pandemic H1N1 influenza infection in China
Source: BMC Infect Dis. 2012 Feb 1;12:29. doi: 10.1186/1471-2334-12-29 (PMC3311613; doi:10.1186/1471-2334-12-29)
Supplement: Additional file 2 — Maternal and neonatal outcomes by different delivery methods in different trimesters. Data are presented as no. (%)/total no.(%), if otherwise stated. Percentages are based on patients with complete information in the respective categories. * Two patients missed the detailed information in maternal outcomes. Neonatal outcomes were unknown in four cases. ** One patient missed the detailed information in maternal outcomes. Neonatal outcomes were unknown in two cases. [file 1471-2334-12-29-S2.DOC]

**Additional file 2 - Maternal and neonatal outcomes by different delivery methods in different trimesters¶.**

| **Variables** | **Preterm delivery (< 37 weeks)**  **(n = 122)*** | | **Non-premature delivery (≥ 37 weeks)**  **(n = 89)**** | |
| --- | --- | --- | --- | --- |
|  | **Vaginal delivery**  **(n = 28)** | **Cesarean delivery**  **(n = 92)** | **Vaginal delivery**  **(n = 8)** | **Cesarean delivery**  **(n = 80)** |
| Maternal outcomes |  |  |  |  |
| Survive | 23/28 (82.1) | 72/92 (78.3) | 6/8 (75.0) | 61/80 (76.3) |
| Death | 5/28 (7.0) | 20/92 (21.7) | 2/8 (25.0) | 19/80 (23.8) |
| Neonatal outcomes |  |  |  |  |
| Survive | 2/28 (7.1) | 66/90 (73.3) | 5/8 (62.5) | 71/79 (89.9) |
| Death | 26/28 (92.9) | 24/90 (26.7) | 3/8 (37.5) | 8/79 (10.1) |

**NOTE:** ¶ Date are presented as no. (%) / total no.(%), if otherwise stated. Percentages are based on patients with complete information in the respective categories.

* Two patients missed the detailed information in maternal outcomes. Neonatal outcomes were unknown in four cases.

** One patients missed the detailed information in maternal outcomes. Neonatal outcomes were unknown in two cases.
